# Simultaneous Biosynthesis of Cellulase by *Aspergillus niger* and *Trichoderma reesei* and Textile Waste Recycling

**DOI:** 10.1007/s12649-026-03488-0

**Published:** 2026-01-29

**Authors:** Etini Etuk, Ali Nawaz, Ziyao Liu, Carol Sze Ki Lin, Chenyu Du

**Affiliations:** 1 School of Applied Sciences, University of Huddersfield, Huddersfield, HD1 3DH UK; 2 School of Energy and Environment, City University of Hong Kong, Kowloon, Hong Kong

**Keywords:** Circular economy, Submerged fermentation, Solid state fermentation, Cellulolytic hydrolysis, Upcycling approach

## Abstract

Textile waste represents a major global challenge due to its substantial contribution to environmental pollution. This study investigated an innovative method for valorising textile waste into cellulase, recycled fibres, and reducing sugars. Various compositions of textile waste, including 100% cotton, 60% cotton blended with 40% Polyethylene terephthalate (PET) and 100% PET, were evaluated for their suitability as substrates in submerged and solid-state fermentation processes aimed at cellulase production using *Aspergillus niger* and *Trichoderma reesei*. The results revealed that maximum cellulase production of 4.75 ± 0.11 U/mL by *A. niger* and 2.41 ± 0.06 U/mL by *T. reesei* was achieved using submerged and solid-state fermentation, respectively. After fermentation, waste fabric was used as substrate for enzymatic hydrolysis into short cotton and PET fibres for recycling purposes. The hydrolysate was analysed for separation and recyclable fibres yield. Cellulase from *T. reesei* (TCEL) demonstrated superior performance compared to cellulase form *A. niger* (ACEL) and commercial cellulase (PAKCEL) by achieving the highest separation yield of 87.0% and a recyclable fibres yield of 68.6%. This comprehensive and novel approach aims to replace today’s linear ‘take-make-dispose’ approach with circular processes, which in turns enable a closed-loop, sustainable textile management.

**Graphical abstract:**

**Figure:**
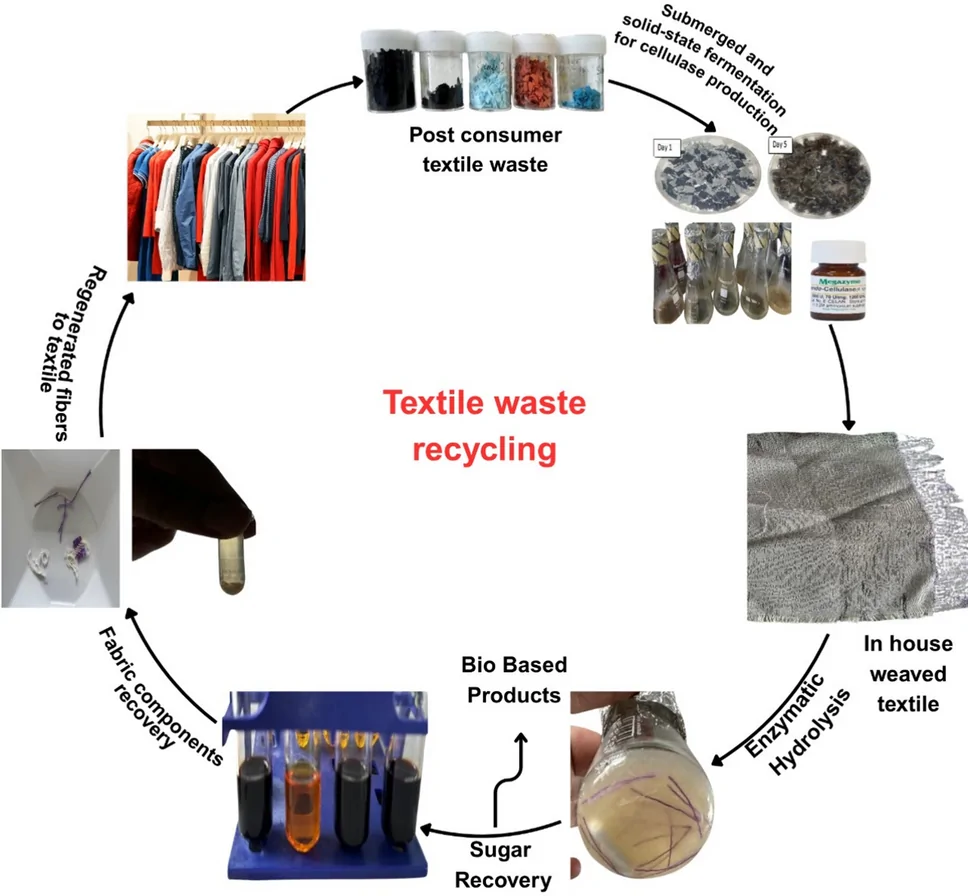

## Introduction

The worldwide demand for textile goods is steadily increasing, with the market valued at USD 1,837.27 billion in 2023 and projected to expand at a compound annual growth rate of 7.4% in revenue from 2024 to 2030 [1]. Countries in Europe, China, the United States, the United Kingdom (UK), and India are significant contributors to textile waste, generating approximately 46 million tonnes of it each year [2]. The textile industry is considered as the second most environmentally harmful sector worldwide after transportation [3] and the fifth-largest source of carbon emissions, responsible for approximately 10% of global carbon emissions [4]. On a global scale, less than 1% of textile waste is recycled into new garments, with 25% being reused or recycled for non-garment applications, whereas approximately 75% ends up in landfill [5]. By 2021, the global output of chemical fibres reached approximately 88.3 million metric tonnes, which included 8.9 million tonnes of synthetic fibres and 7.4 million tonnes of cellulose fibres [6]. The increasing use of synthetic fibres in the textile industry also significantly contributes to micro-plastic pollution as broken fibres are released into the water system during washing [7].

Moreover, the textile sector significantly affects the water footprint, marked by high water usage in production, water contamination, and the considerable water requirements of cotton farming [8]. For example, producing a single cotton T-shirt requires between 2,500 and 3,000 L of water [9]. Additionally, wastewater from this industry may contain hazardous heavy metals such as antimony, lead, arsenic, chromium, cadmium, and mercury, along with various aromatic compounds and pesticides. These enduring pollutants can travel through and pollute waterways and oceans, positioning textile production as a major source of serious water pollution issues [10]. Consequently, the industry has begun to transition from the linear economic model of 'take-make-waste' to the circular economy framework, wherein textiles can recycle that leads to resource conservation and reduced environmental impact [11].

The challenges posed by textile waste have generated considerable interest in its biorefining into valuable products. The recycling of textiles is thought to have beneficial environmental impacts by increasing the availability of virgin textile fibres and shortening the overall life cycle of textile goods [12, 13]. Cotton fibres, primarily composed of cellulose, are especially susceptible to microbial degradation. Research indicates that various microorganisms, including bacteria such as *Cytohaga* spp., *Cellulomonas* spp., *Cellvibrio* spp., *Bacillus* spp., and *Clostridium* spp., as well as fungi such as *Aspergillus* spp., *Alternaria* spp., *Trichoderma* spp., and *Penicillium* spp., can generate cellulolytic enzymes to degrade textile waste [14, 15]. Microbial cellulase presents considerable potential for a range of applications in commercial fields, including textiles, paper and pulp, laundry, brewing, agriculture, and biofuels. Utilising microorganisms with high rates of cellulase production for the degradation of textile waste is a viable process [16]. Additionally, recent studies have focused on the bioconversion of textile waste into fermentable glucose through the pretreatment and hydrolysis of cellulose [17, 18].

Previous research has demonstrated the utilization of textile waste for cellulase production through both solid-state and submerged fermentation. Various strains of *Aspergillus niger* and *Trichoderma reesei* were employed to valorise polycotton textile waste, achieving a cellulase activity of 1.18 ± 0.05 FPU/g via solid-state fermentation [19]. Further optimization of solid-state fermentation conditions resulted in a 25.8% increase in cellulase yield [20]. The produced cellulase was benchmarked against commercial cellulase, attaining a 70% hydrolysis yield after 96 h, with cellulose converted to glucose and synthetic fibres recovered. Similarly, submerged fermentation using different strains of *A. niger* and *T. reesei* was evaluated for cellulase production. Application of the resulting cellulase, compared to commercial cellulase, achieved 41.6% cellulose-to-glucose conversion and recovery of synthetic fibres after 96 h of textile hydrolysis [21]. Other research groups primarily concentrate on hydrolysing textile waste using commercial cellulase, rather than utilizing textile waste first for production and subsequently as a substrate for hydrolysis. Cho et al. [22] investigated textile hydrolysis to produce value-added chemicals such as bioethanol, lactic acid, and sorbitol. Similarly, Boondaeng et al. [23] demonstrated the hydrolysis of polycotton textiles using commercial enzymes, achieving an 84% hydrolysis rate with glucose as the main product.

Despite considerable efforts, research gap lies in finding a sustainable, cost-effective, and integrated strategy for valorising textile waste, rather than merely utilising it for partial product such as glucose and associated fermented chemicals recovery remains elusive. Therefore, this study focused on finding a cost-effective way of producing cellulase through comparative assessment of submerged and solid-state fermentation for fungal strains, then utilize the enzymes for textile waste recovery. The novelty of this study lies in its focus on recovering recyclable fibres—both cotton and PET—rather than converting cellulose into glucose. This is achieved through a short-term hydrolysis process lasting 4 h, in contrast to the extended 96-h hydrolysis methods employed in our previous work [19–21] and reported by other research groups [22, 23]. Furthermore, textile hydrolysis in this study was performed on in-house woven fabric composed of white cotton fibres and purple PET fibres, which were intentionally dyed to facilitate fabric recovery analysis. Two new terms of separation yield (reducing sugars, short chain cotton residues and separated cotton and PET fibres) and recyclable fibres yield (short chain cotton residues and separated cotton and PET fibres) were introduced for first time in this study for establishing the integrated biorefinery approach to make textile waste recycling process truly circular by expanding the scope of sustainability, to in turn enable development towards a closed-loop textile industry.

Thus, the present study adopts the novel approach of co-producing cellulase, recycled fibres, and fermentable sugars via the hydrolysis of textile waste by investigating the capabilities of various fungal strains to produce cellulase while utilising textile waste as a substrate to facilitate the recycling of virgin fibres.

## Materials and Methods

### Microorganisms

The fungal strains i.e. *Aspergillus niger*, *Aspergillus oryzae*, *Aspergillus awamori* and *Trichoderma reesei* used in this study were sourced from Professor Du's laboratory at the University of Huddersfield, UK [19]. These strains were assessed for cellulase production, by growing on PDA supplemented with 0.5% carboxymethyl cellulose (CMC) and 0.1% Congo red at 30 °C for 48 h [24]. Following incubation, the plates were examined for hydrolytic zones surrounding the cellulase-producing fungal strains. The cellulolytic fungi were then cultivated by submerged fermentation for the quantification of the produced cellulase [24].

### Textile Waste Pretreatment

Pretreatment of post-consumer textile waste alter the fibre structure and enhance the accessibility of key components (e.g., cellulose) to fungi for better fermentation performance. The post-consumer textile waste i.e. 100% cotton, 100% PET, blend of 60% cotton and 40% PET and in house weaved fabric i.e. blend of 84% cotton and 16% PET were cut into 1 × 1 cm^2^ squares using industrial-grade scissors and placed in a 100-mL Duran bottle for treatment with a solution containing NaOH (7% w/v) and urea (12% w/v) at a solid-to-liquid ratio of 1:20 (w/v). The fabric was allowed to immense into the solution for 15 min at room temperature while being agitated with a magnetic stirrer bar, after which it was frozen at -20 °C for 6 h [25]. Thereafter, the samples were removed from the freezer, allowed to return to room temperature, thoroughly washed with tap water, and then air-dried. This pretreated post-consumer fabric was used as substrate for cellulase production. Whereas, pretreated in house weaved fabric i.e. blend of 84% cotton and 16% PET was used for enzymatic hydrolysis. Cotton and PET fibres were dyed white and purple purposely in In-house weaved fabric to detect the separation easily during enzymatic hydrolysis.

### Fermentation

Cellulase production by fungal strains such as *A. niger* and *T. reesei* while utilising pretreated post-consumer textile waste as the substrate was examined via both submerged and solid-state fermentation methods.

#### Inoculum Preparation

 The spore inoculum was prepared using sterilized normal saline (0.85% NaCl) solution of deionized waste. The normal saline was added in the fungal slants aseptically. The spores at the surface of the slants were gently rubbed off in the normal saline. The spore suspension was homogenized by gentle mixing using vortex machine. The spore suspension was serially diluted to get the spore count of 1.5 × 10^7^ spores/mL (counted using Neubauer chamber).

#### Submerged Fermentation

Post consumer textile (0.5g) was added to 50 mL of fermentation medium (composition per 500 mL: cellulose powder 1 g, urea 0.15 g, KH_2_PO_4_ 1 g, (NH_4_)_2_SO_4_ 0.7 g, MgSO_4_ 0.15 g, CaCl_2_ 0.2 g, yeast extract 2.5 g, FeSO_4_ 0.5 g, MnSO_4_ 0.16 g, ZnSO_4_ 0.14 g, CoCl_2_ 0.2 g) in a 250-mL conical flask. Wheat straw and *Miscanthus* (0.5 g) were used as natural reference substrates, and fermentation medium without substrate was used as a control. After sterilisation, the fermentation media were aseptically inoculated with 0.1 mL of fungal spore suspension (1.5 × 10^7^ spores/mL) and incubated at 30°C for 5 days in a shaking incubator at 180 rpm. The fermentation broth was analysed for cellulase activity after 3 and 5 days of incubation.

#### Solid-state Fermentation

Solid-state fermentation was performed at a solid-to-liquid ratio of 1:6, i.e., by adding 8 g textile waste to 48 mL of diluent (composition [g L^−1^]: urea, 0.3; KH_2_PO_4_, 2; (NH_4_)_2_SO_4_, 1.4; MgSO_4_, 0.3; CaCl_2_, 0.4; FeSO_4_, 0.005; MnSO_4_, 0.0016; ZnSO_4_; 0.0014; CoCl_2_, 0.002) in reagent bottles. After autoclaving for 15 min, the samples were inoculated with 0.3 mL spore suspension ((1.5 × 10^7^ spores/mL) of *A. niger* and *T. reesei* and mixed for thorough distribution. The inoculated textile samples were then spread on petri plates and incubated at 30°C for 7 days. After incubation, textile waste (substrate) was transferred into 50 mM sodium citrate buffer (pH 4.8) and shaken at 50 rpm for 60 min to recover the enzymes. Subsequently, the solid substrate was filtered out of the buffer using muslin cloth and enzyme recovered in buffer was subjected to cellulase estimation.

### Enzyme Assay

#### Cellulase Activity

Cellulase activity was quantified in filter paper units (FPU) following the methodology established by Ghose [26]. A crude extract of 0.5 mL was combined with 0.5 mL of 50 mM citrate buffer at pH 4.8. A 1 × 6 cm Whatman No. 1 paper strip, weighing approximately 50 mg and serving as the cellulose substrate, was introduced into the tube and incubated at 50°C for 30 min. Subsequently, 3 mL of dinitro salicylic acid (DNS) reagent, prepared following the procedure reported by Ghose [26], was added to each tube, and the mixture was boiled for 10 min. After cooling with ice for 5 min, the samples were diluted (100 µL of sample mixed with 900 µL of distilled water), and the absorbance was measured at 540 nm using a spectrophotometer. The FPase activity was calculated using the following equation:

$$FPase activity \left(\frac{U}{mL}\right)=\frac{0.37}{concnetration\; of\; enzyme\; that\; release\; 2\; mg\; Glucose}$$

#### Endoglucanase, Exoglucanase, and Glucosidase activity

The individual enzymatic activity or cellulase components (endoglucanase, exoglucanase, and glucosidase) were estimated following the procedure adopted in our previous study [19] using CMC, Avicel and *p*-nitrophenyl-β-d-glucopyranoside as specific substrates, respectively. The enzyme activity for endoglucanase, exoglucanase and glucosidase was calculated using the method developed by Ghose [26] and Herr [27], respectively.

### Enzymatic Hydrolysis

The recovered enzymes from A. niger (ACEL) and T. reesei (TCEL) were added at a concentration of 23 FPU to a conical flask containing 2.5 mL of 0.5 M sodium citrate buffer (10 ×) to facilitate enzymatic hydrolysis of 0.5 g of textile waste (TWILL 12, comprising 84% cotton and 16% PET). The total reaction volume was adjusted to 50 mL using distilled water. The commercial enzyme PAKCEL was used at the same concentration for comparison. The hydrolysis was performed at 50°C and 300 rpm for 4 h. After hydrolysis, the remaining textile fabric and separated textile fibres were recovered by passing the reaction mixture through sieve (10 mm pore size). The hydrolysate still with short chain cotton residues was subjected to centrifugation at 6,000 rpm for 10 min. The short chain cotton residues were recovered as pellet. The supernatant was analysed for presence of reducing sugars by high performance anion-exchange chromatography (HPAEC) (Dionex IC 2500) on a Dionex Carbopac PA-10 column with integrated amperometry detection [28]. A control was run in parallel having all the components like experimental flasks except enzyme. The dry weights of cotton yarn, plastic fibres, and short chain cotton residues were recorded, and filtrate was analysed for the concentration of reducing sugars. The separation yield was calculated as cumulative recovery yield reducing sugars, short chain cotton residues, separated cotton and PET fibres. Recyclable fibre yield was calculated as cumulative recovery yield of short chain cotton residues and separated cotton, PET fibres.

### Statistical Analysis

All the experiments were conducted in triplicate, and the results are presented as the means ± standard deviation. Paired, two-tailed Student’s t-test was performed using Excel to assess statistical significance.

## Results and Discussion

### Comparative Cellulase Activity

**Fig. 1:**
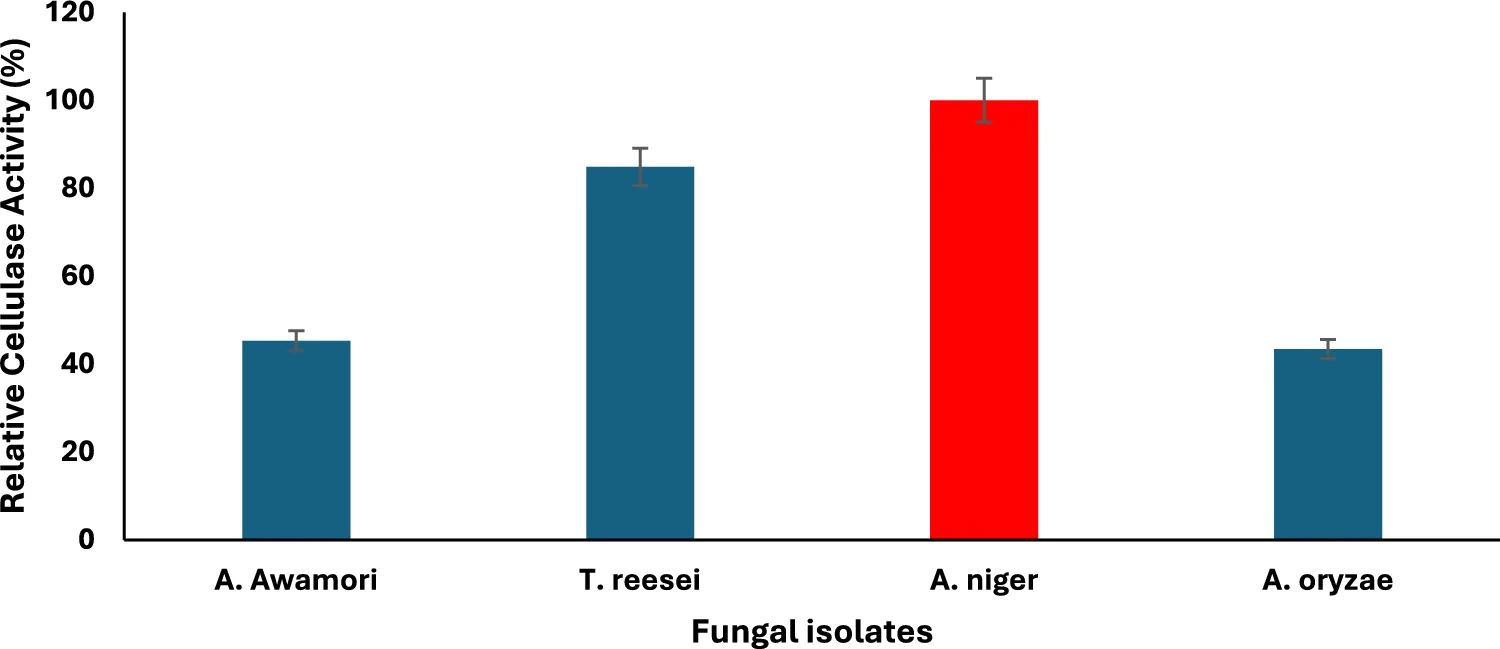
Comparative analysis of the potential of different fungal strains to produce cellulase via submerged fermentation using 0.5% CMC as sole carbons source. The red bar indicates the cellulase activity of *A. niger*, with 100% corresponding to 0.109 ± 0.02 U/mL. Reaction conditions: Temperature 30 °C; Agitation speed; 180 rpm; Incubation time: 72 h. Data are shown as mean ± SE; error bars represent standard error (n = 3 replicates)

Four fungal strains (*A. niger*, *A. oryzae*, *A. awamori*, and *T. reesei*) that were already available in the laboratory were assessed for their cellulase production via submerged fermentation [21]. *A. niger*, *A. oryzae*, *A. awamori*, and *T. reesei* are prominent fungal species widely utilized for cellulase production in industries such as biofuels and textiles [29]. While *T. reesei* typically achieves higher enzyme yields, *Aspergillus* species are valued for their complete cellulase systems and robust overall enzyme secretion [30]. The results indicated that *A. niger* and *T. reesei* exhibited substantially higher cellulolytic activity compared to the other strains, with relative activities of 100% (0.109 ± 0.002 U/mL) and 84.9% (0.092 U/mL), respectively. In contrast, the remaining strains produced significantly lower levels of cellulase than these two species (Fig. 1). A similar pattern was reported by Septiani et al. [31], who observed a 19.8% advantage of *A. niger* (0.131 U/mL) over *T. reesei* (0.106 U/mL) when using 1% CMC as the sole carbon source, compared to 0.5% CMC in the present study. Likewise, Roy et al. [32] documented cellulase production of 0.893 ± 0.0018 U/mL and 0.872 ± 0.0013 U/mL by *Aspergillus* sp. DAJ2 and *Trichoderma* sp. DTJ4, respectively. The comparatively higher enzyme activities in these studies may be attributed to the use of CMC as the substrate in the enzyme assay, rather than filter paper as employed in the present work and by Septiani et al. [31]. The superior cellulase production observed in *A. niger* and *T. reesei* can be attributed to their well-established role as major cellulase producers and their extensive application in the industrial production of various enzymes [33]. Consequently, these two species were selected for further experimentation.

### Cellulase Production

#### Submerged Fermentation

**Fig. 2:**
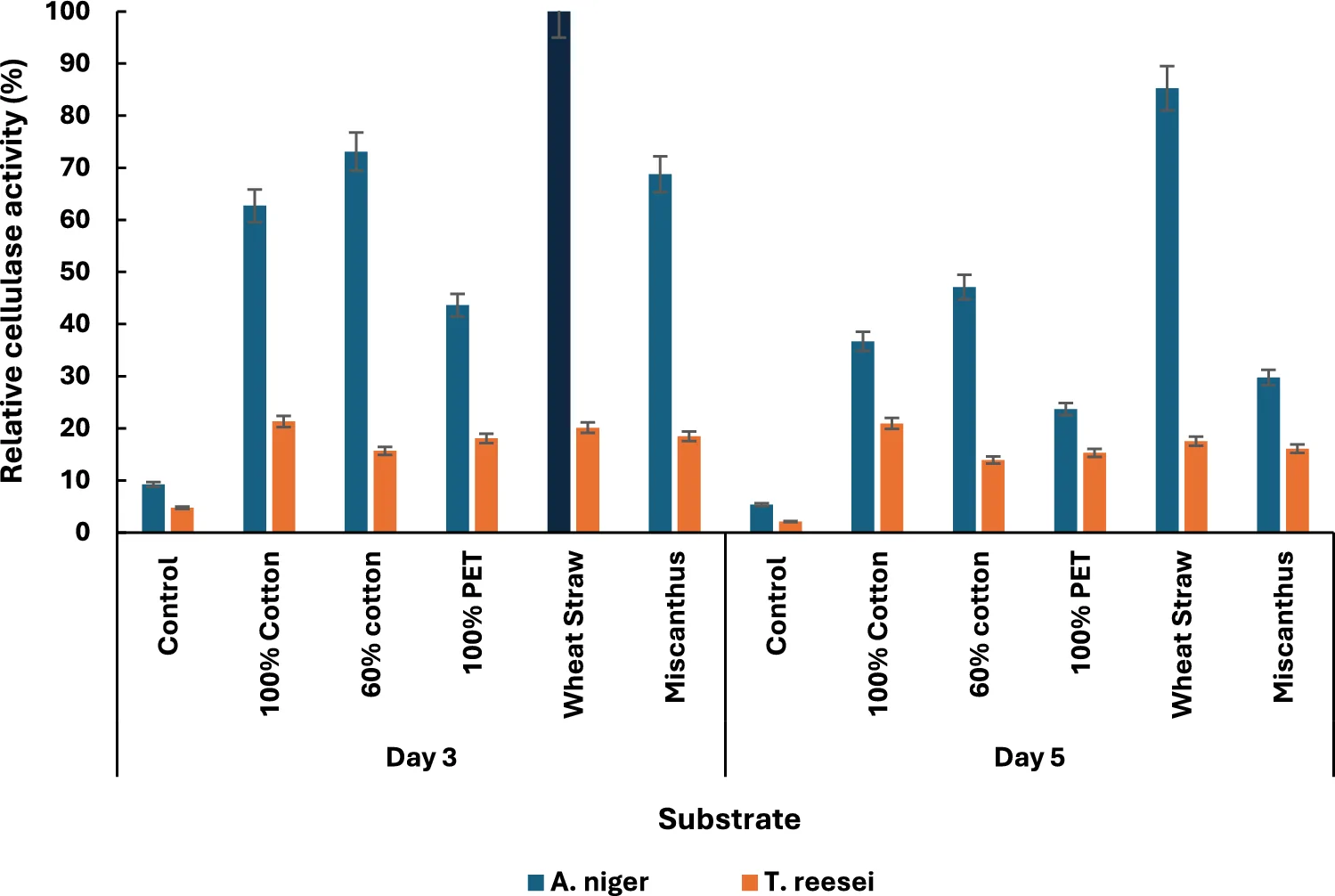
Comparative analysis of cellulase activity by *A. niger* and *T. reesei* using textile waste and natural substrates in submerged fermentation*.* The dark blue bars indicate the cellulase activity of *A. niger* on wheat straw, with 100% corresponding to 4.75 ± 0.11 U/mL. Reaction conditions: Temperature 30 °C; Agitation speed; 180 rpm. Data are shown as mean ± SE; error bars represent standard error (n = 3 replicates)

The potential for *A. niger* and *T. reesei* to produce cellulase by utilising different compositions of textile waste as substrates (100% cotton, 60% cotton + 40% PET, and 100% PET), organic substrates such as wheat straw and *Miscanthus*, and control with no substrate was evaluated via submerged fermentation over 3 and 5 days of incubation. The findings demonstrated that *A. niger* outperformed *T. reesei* in cellulase production rate across all substrates after 3 days of incubation (Fig. 2). Continued incubation to 5 days has resulted in decreased cellulase production across all substrates. The decline in cellulase production after extended incubation is likely due to nutrient depletion, accumulation of inhibitory metabolites, pH shifts, and enzyme degradation over time, as cultures transition from active growth to the stationary phase [34]. Among the substrates tested, *A. niger* showed the highest relative cellulase activity of 100% with wheat straw, followed by cellulase activity of 73.1% with textile waste (60% cotton + 40% PET), 68.8% with *Miscanthus*, 62.8% with cotton, and 43.6% with PET after 3 days of incubation. Importantly, in the absence of any inducers, cellulase production by *A. niger* was restricted to 9.2%, highlighting the essential role of inducers in the activation of genes that govern cellulase synthesis. The results clearly indicate that textile waste can be utilized as a viable alternative substrate for cellulase production, building upon our earlier findings on cellulase synthesis via submerged fermentation using multiple strains of *A. niger* and *T. reesei*, where maximum cellulase activity of 7.51 FPU/g was attained using polycotton blend (60% cotton + 40% PET) using *A. niger* HDU [21]. A similar trend was observed in this study, where the cotton 60%–PET 40% blend exhibited slightly higher cellulolytic activity—1.06 times greater than that of pure cotton—likely due to the supportive surface provided by PET, which facilitates effective cellulose degradation by the fungi. However, cellulolytic activity on both the cotton–PET blend and pure cotton was markedly higher than on PET alone—by 1.67- and 1.58-fold, respectively—owing to the presence of accessible cellulose that fungi can readily metabolize. This work incorporates fermentation results using wheat straw and *Miscanthus* as natural reference substrates, along with a control medium without any substrate, to strengthen the validity of the findings. There are no more reports available in literature to compare results of the current research, to best of our knowledge, on valorisation of textile waste as substrate for cellulase production by submerged fermentation.

### Solid-state Fermentation

**Fig. 3:**
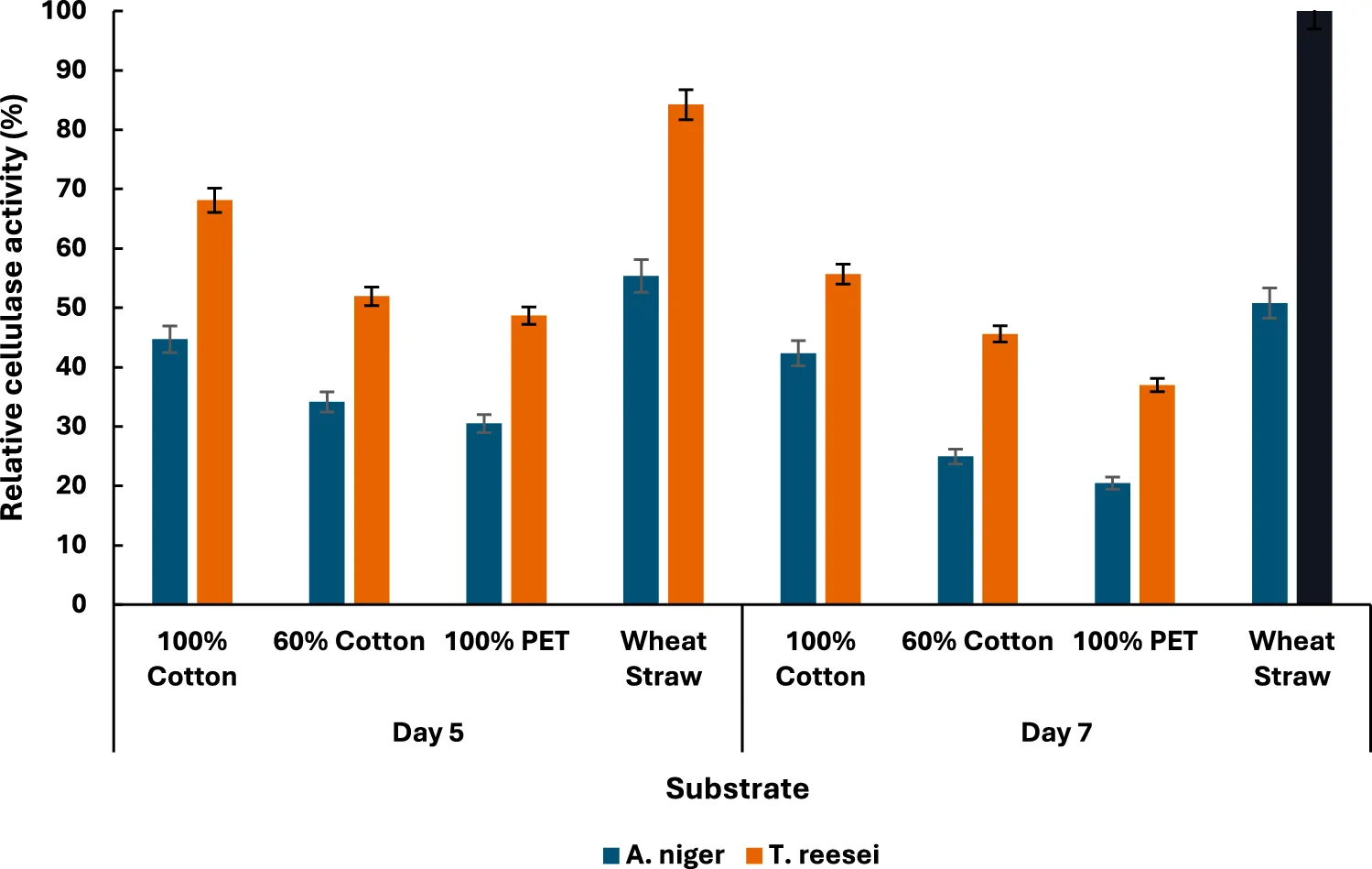
Comparative analysis of cellulase production by *A. niger* and *T. reesei* using textile waste and wheat straw in solid-state fermentation*.* The dark blue bars indicate the cellulase activity of *T. reesei* on wheat straw, with 100% corresponding to 2.41 ± 0.06 U/mL. Reaction conditions: Temperature 30 °C and solid-to-liquid ratio of 1:6. Data are shown as mean ± SE; error bars represent standard error (n = 3 replicates)

The potential for *A. niger* and *T. reesei* to produce cellulase by utilising textile waste (100% cotton, 60% cotton + 40% PET, and 100% PET) and wheat straw as substrates was also evaluated via solid-state fermentation (SSF) over 5 and 7 days of incubation. As illustrated in Fig. 3, *T. reesei* exhibited the highest cellulase production, achieving a relative activity of 100% after 7 days of incubation, whereas *A. niger* demonstrated 55.4% cellulase activity after 5 days when using wheat straw as the substrate. The observed differences in substrate utilisation rates among the fungal strains may be attributed to differences in their metabolic activity and mechanisms [35]. *T. reesei* is widely recognized for its highly efficient cellulolytic enzyme complex, comprising synergistic endoglucanases, exoglucanases, and β-glucosidases, which collectively enable comprehensive cellulose hydrolysis [36]. In contrast, *A. niger* exhibits a broader enzymatic profile, with pronounced hemicellulase and pectinase activity but comparatively lower β-glucosidase activity, thereby limiting its capacity for complete cellulose breakdown [37]. Additionally, regulatory factors play a critical role; *T. reesei* demonstrates robust induction of cellulase gene expression in response to cellulose, sustaining enzyme secretion over extended incubation periods, whereas *A. niger* is more susceptible to catabolite repression, which curtails cellulase synthesis upon accumulation of simple sugars [38, 39]. In contrast, when utilizing textile waste substrates, *T. reesei* exhibited the highest enzyme activity across all tested materials, achieving its best yield of 68.1% after 5 days of incubation with pure cotton. Conversely, cellulase production using a 60% cotton–40% PET blend and 100% PET was 1.31-fold and 1.39-fold lower, respectively, under the same incubation conditions (Fig. 3). The higher cellulase yield observed after 5 days with textile substrates compared to wheat straw may be due to the simpler structural composition of textiles, which allows the fungi to metabolize them more efficiently in a shorter time. In our previous study, we conducted a preliminary assessment of solid-state fermentation (SSF) using *A. niger* and *T. reesei* with textile waste as the substrate for cellulase production. The results indicated that *A. niger* was the superior cellulase producer, achieving 1.18 ± 0.05 FPU g⁻^1^ with an 80% cotton–20% PET blend [19]. Subsequent optimization studies further improved cellulase activity to 1.56 ± 0.03 FPU g⁻^1^ [20]. However, these investigations did not evaluate the potential of *T. reesei* under optimized conditions in comparison to *A. niger*, which is the focus of the present study. Additionally, wheat straw was investigated as a natural substrate alongside textile materials to compare their respective roles in cellulase production—a comparison that had not been addressed in our previous studies. Currently, there are no additional reports that have investigated textile waste as a substrate for cellulase production through fungal fermentation.

### Enzyme Profile

**Fig. 4:**
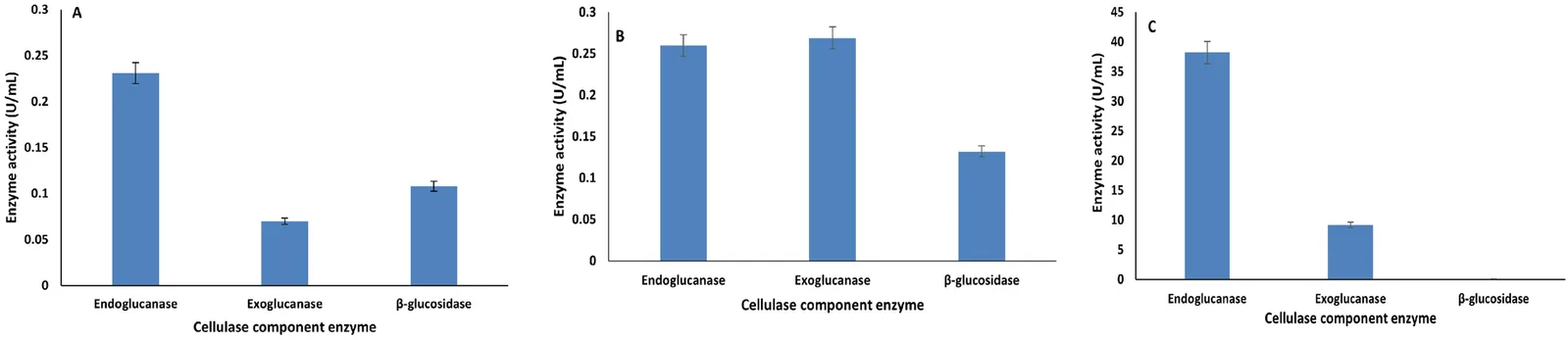
Enzyme profiling (Endoglucanase (CMCase), Exoglucanase (Avicelase) and β-glucosidase) of the cellulases **A**: ACEL from *A. niger*; **B**: TCEL from *T. reesei*; and **C**; PAKCEL commercial cellulase from Yethious Trading Ltd. respectively. All the values presented are the means ± standard deviations from three separate experiments (n = 3). Data are shown as mean ± SE; error bars represent standard error (n = 3 replicates)

A comparative analysis of cellulase production by *Aspergillus niger* under submerged and solid-state fermentation indicated that cellulase from submerged fermentation was preferred for subsequent experiments due to its shorter production time (3 days) and significantly higher cellulolytic activity—approximately 3.57 times greater than solid-state fermentation. In contrast, *Trichoderma reesei* produced 2.41 ± 0.06 U/mL in solid-state fermentation after 7 days compared to 1.01 ± 0.04 U/mL in submerged fermentation after 3 days; however, submerged fermentation was still favoured because of its reduced production time. The primary obstacle in the production and adaptation of fungal cellulases during solid-state fermentation is poor translocation of nutrients within fungal mycelium, which limit the rate of fungal growth in particulate bioprocessing [40]. These limitations may result in increased production cost and the reduced viability of scaling up [41]. The cellulase enzymes were determined to evaluate the relative amounts of endoglucanase, exoglucanase, and β-glucosidase prior to utilisation in hydrolysis of textile waste. Such profiling is essential to obtain an in-depth insight into the structural and functional characteristics of enzymes and thus guide their optimisation in their subsequent applications [42]. The observed enzymatic activity profiles of ACEL and TCEL indicate notable differences in the distribution of cellulase components, with ACEL exhibiting endoglucanase (CMCase), exoglucanase (avicelase), and β-glucosidase activities of 0.231 ± 0.04 U/mL, 0.07 ± 0.02 U/mL, and 0.108 ± 0.01 U/mL, respectively, while TCEL demonstrated corresponding activities of 0.260 ± 0.03 U/mL, 0.269 ± 0.04 U/mL, and 0.132 ± 0.04 U/mL (Fig. 4). These variations suggest that the two fungal species employ distinct strategies for cellulose degradation, likely driven by genetic and regulatory differences that govern enzyme synthesis and secretion. For instance, *T. reesei* is known for its highly coordinated cellulolytic system, which emphasizes synergistic action between exoglucanases and endoglucanases, whereas *A. niger* typically exhibits a broader hydrolytic profile with comparatively lower β-glucosidase activity [43]. PAKCEL was mainly composed of endoglucanase and exoglucanase with CMCase, avicelase, and β-glucosidase activities of 38.2 ± 0.05 U/mL, 9.2 ± 0.01 U/mL, and 0.132 ± 0.03 U/mL. Sulyman et al. [44] reported an endoglucanase to exoglucanase ratio of 3:1, which contrasts with the findings of this study, potentially due to the different genetic expression of the *A. niger* strains used. Conversely, Silva et al. [45] documented an endoglucanase to exoglucanase activity ratio of 1:1 in *T. reesei*, which aligns with the results of this study. This indicates that fungal cellulase systems can exhibit diverse ratios of endoglucanases to cellobiohydrolases, thereby influencing the efficiency of cellulose degradation [46].

### Textile Hydrolysis

**Fig. 5:**
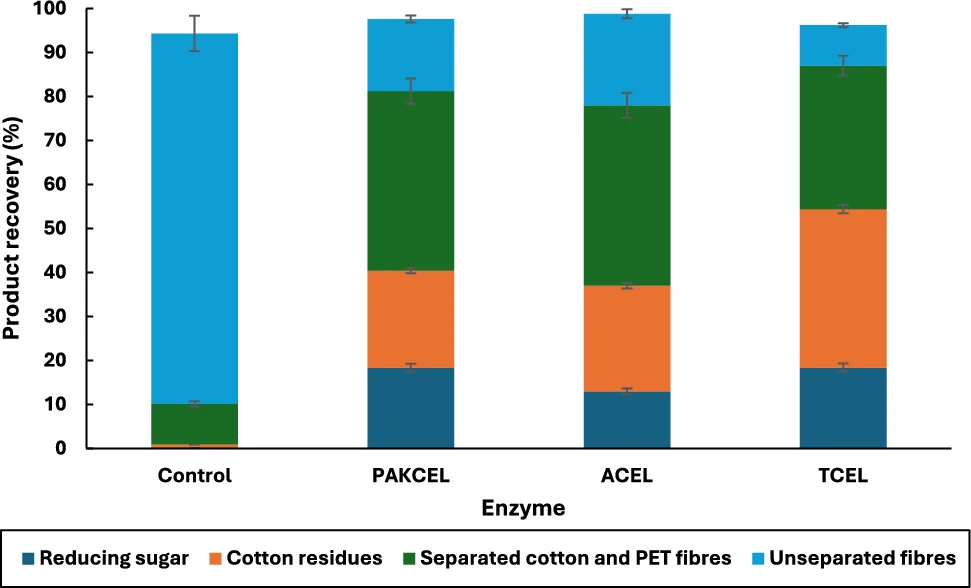
Textile hydrolysis profile using in-house produced and commercial cellulases. Enzyme loading of 50 FPU/g of fabric was used. Reaction conditions: volume 50 mL, temperature 50°C, agitation speed 300 rpm and incubation time 4 h. Data are shown as mean ± SE; error bars represent standard error (n = 3 replicates)

**Fig. 6:**
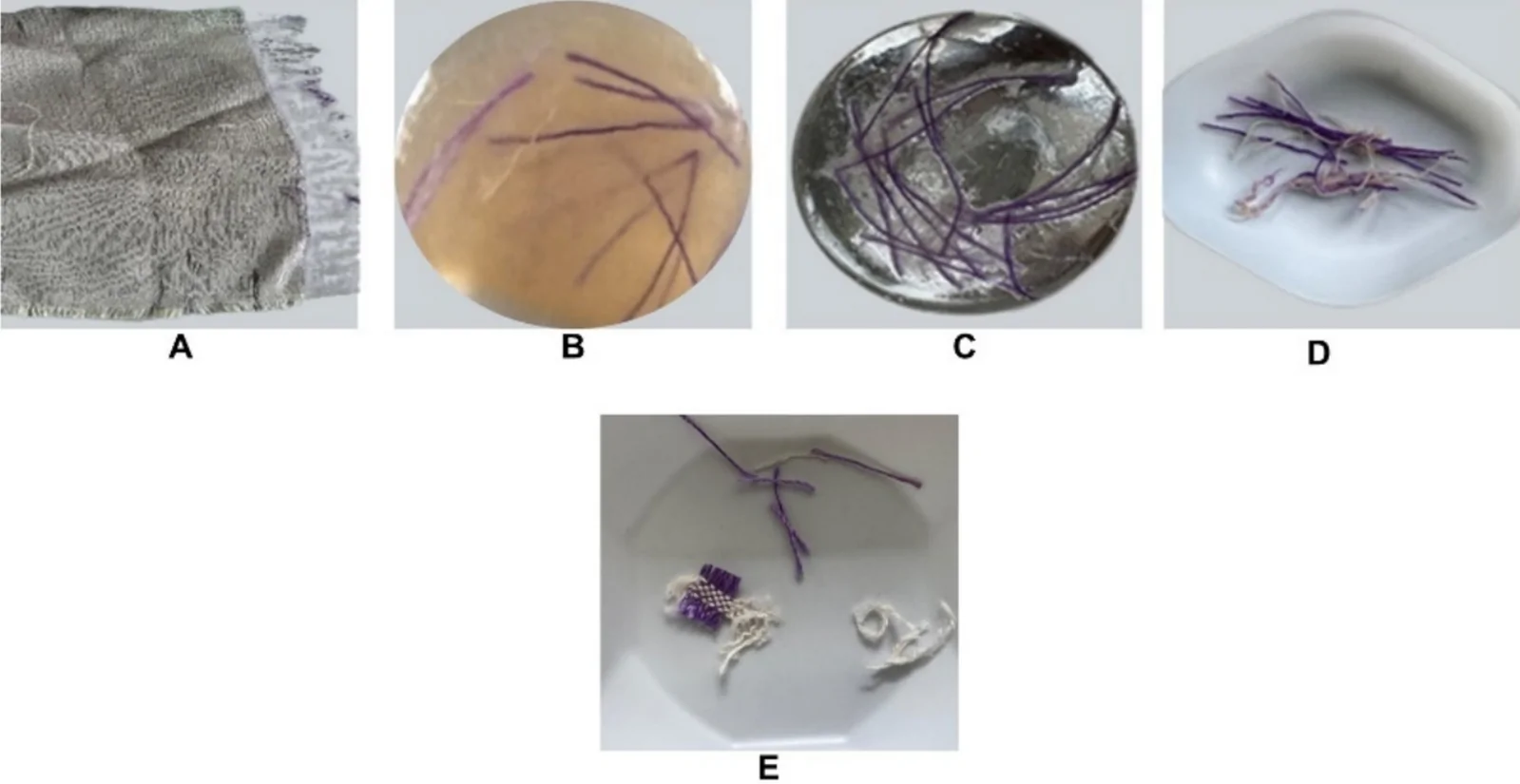
Pictorial journey of textile hydrolysis: **A** = In-house produced fabric TEILL 12, 84% cotton and 12% PET; **B** = Enzymatic hydrolysis in a conical flask; **C** = post-hydrolysis separated fabric in a flask; **D** = Wet separated fabric after recovery; **E**: Dried separated cotton (white) and PET (purple) fibres along with unseparated flakes

A comparative analysis of the in-house produced cellulases ACEL and TCEL and the commercial cellulase PAKCEL was conducted to evaluate their efficacy in hydrolysing lab-woven textile TWILL 12 composed of 84% cotton and 12% PET. The overall hydrolysis recovery rates were found to be similar, with PAKCEL achieving a 97.6% recovery rate, ACEL 98.9%, and TCEL 96.2% (Fig. 5). The recovery of reducing sugars was nearly identical for PAKCEL and TCEL, at 18.36% and 18.4%, respectively, whereas that by ACEL was lower at 13%. The highest recovery of cotton residues was 36% with TCEL, followed by 24% with ACEL and 22.04% with PAKCEL. The high recovery with TCEL may be attributable to its high exoglucanase concentration. In terms of separated cotton and PET fabric recovery, the highest recovery was 41% with ACEL, followed closely by 40.8% with PAKCEL and 32.6% with TCEL. The unseparated fabric flakes recovery with ACEL, PAKCEL, and TCEL was 20.8%, 16.4%, and 9.2%, respectively. These findings suggest that ACEL performs comparably to commercial enzymes in obtaining intact separated cotton and PET fibres which are suitable for recycling. In the past decade, research have been mainly focused on converting cotton in textile blends to sugars and recovering the polyester for recycling. Valentukeviciene et al. [47] and Simonetti et al*.* [48] reported sugar recovery rates of 30% and 94.6%, respectively, both of which exceed the yield observed in this study. This difference may be attributable to their prolonged incubation period of 2 days and 1 day, respectively, whereas only 4 h was applied in this research. A sugar recovery of 18.4% after 4 h of incubation period in the current study is commendable, as compared with the 30% reported by Valentukeviciene et al. [36] after 2 days of incubation. The high sugar recovery of 94.6% reported by Simonetti et al. may be attributable to an adverse mechano-chemical pretreatment before hydrolysis. The focus of textile valorisation has rapidly transitioned from the extraction of sugars for textile recycling and related applications to the fractionation of fibres. Villar et al. [49] attained a fabric recovery rate of 65% through hydrolysis coupled with mechanical separation, a result that is notably close to the 60% yield achieved with ACEL without mechanical separation support. In summary, TCEL demonstrated superior performance compared to ACEL and PAKCEL by achieving the highest separation yield of 87% (comprising the total of reducing sugar, cotton residues, and the recovery of separated fibres), and a recyclable fibres yield of 68.6% (which includes the total of cotton residues and the recovery of separated fibres) as evident from Table 1. Furthermore, Fig. 6 presents the different steps of enzymatic hydrolysis to achieve the hydrolysis and separation of textile fabric.

**Table 1:** Separation and recyclable fibres yield analysis of textile hydrolysis by different cellulase

Sr. No	Enzyme	Separation yield (%) *	Recyclable fibres yield (%) **
1	Control	10.1	10.0
2	ACEL	78.0	65.0
3	TCEL	87.0	68.6
4	PAKCEL	81.2	62.84

^*^Separation yield is the sum of reducing sugar, cotton residues and separated fibres recovery

^**^Recyclable fibre yield is the sum of cotton residues and separated fibres recovery

## Conclusions

Textile waste is an excellent substrate for the fungal biosynthesis of cellulase via submerged and/or solid-state fermentation. A mixture of cotton and PET (60:40) was found to be 1.06 times more effective for cellulase production than pure cotton or PET alone. Enhanced cellulase production by *A. niger* via submerged fermentation and by *T. reesei* via solid-state fermentation demonstrated that the choice of fungal strain and fermentation method are vital factors influencing the yield of cellulase. Cellulases produced by different fungal species exhibit varying efficiencies in hydrolysing textile materials due to their varying subunit compositions. Remarkably, in-house produced enzymes demonstrated similar effectiveness to a commercial cellulase in hydrolysing textile waste. Furthermore, the development of novel textile waste recycling strategy for production of fermentable sugars as carbon source for fermentative production of value-added products in turn would contribute to realising the circular economy and securing our sustainable future by addressing the United Nations Sustainable Development Goals. Enzymatic recycling of textile fibres offers significant opportunities for sustainable material recovery. Hydrolysed fibres can be converted into fermentable sugars for bioethanol production, bioplastics, and organic acids, supporting green manufacturing. Additionally, recovered cellulose can be repurposed into nanocellulose for advanced composites, packaging, and biomedical applications. This approach aligns with circular economy principles and contributes to reducing textile waste in landfills.

## Data Availability

Data available as supplementary information.
